# Novel multifunctional cheese-like 3D carbon-BN as a highly efficient adsorbent for water purification

**DOI:** 10.1038/s41598-018-19541-5

**Published:** 2018-01-18

**Authors:** Zhenya Liu, Yi Fang, Huichao Jia, Chong Wang, Qianqian Song, Lanlan Li, Jing Lin, Yang Huang, Chao Yu, Chengchun Tang

**Affiliations:** 1 School of Materials Science and Engineering, Hebei University of Technology, Tianjin, 300130 P. R. China; 2 Hebei Key Laboratory of Boron Nitride Micro and Nano Materials, Hebei University of Technology, Tianjin, 300130 P. R. China

## Abstract

In this paper, a novel three dimensional carbon boron nitride (3D C-BN) was successfully prepared. The obtained material has porous cheese-like structure and pore size ranging from 2 nm to 100 nm. Attractively, the 3D C-BN, which combines the adsorption advantages of BN and carbon together, exhibits excellent adsorption properties for organic dyes, oils and heavy metal ions. The maximum removal capacities of 3D C-BN for methyl blue (MB) and congo red (CR) are 408 mg g^−1^ and 307 mg g^−1^, respectively. Furthermore, 3D C-BN can quickly and efficiently remove oils (salad oil, gasoline and pump oil) and heavy metal ions (Cr^3+^, Cd^2+^ and Ni^2+^) from waste water. The macro bulk 3D C-BN, which is more convenient to use than powdered materials, can be reused by burning or heating in air and still maintains high adsorption capacity. Significantly, these superior performances can find practical application in water purification.

## Introduction

In recent years, numerous researches have focused on nanomaterials because of their small sizes and high accuracy. Correspondingly, with the rapid update of nanomaterials, our daily life gets more and more advanced and convenient^1,2^. 3D nanomaterials, including 3D metals, 3D ceramics, 3D polymers, have attracted tremendous attention in many fields such as catalysts, recording media, optical materials and fuel cells due to their outstanding properties and potential applications^3–6^. 3D carbon, as a practical nanomaterial, is generally applied to super capacitor, biology and efficient removal materials^7–11^. Recently, Jiang *et al*. fabricated micro cellular 3D graphene foam with a cellular morphology, and its surface-to-volume ratio was 2.5 × 10^5^ m^2^ m^−3^. Moreover, the graphene foam had potential applications in energy storage devices for balanced high loading and fast charging and discharging rates^12^.

Hexagonal boron nitride (h-BN), known as “white graphite”, is an isostructure of carbon which possesses preeminent physical and chemical properties such as low density, high thermal conductivity, chemical stability, as well as the outstanding adsorption properties^13–19^. Lately, 3D BN materials are commonly studied. For example, Lian *et al*. presented a facile solid phase method for preparing unique 3D hBN nanoflowers with good thermal stability and high specific surface area^20^. Zhao *et al*. fabricated 3D BN foam by a vesicant-assisted gas-foaming process. The product had a vesicular structure with hierarchical pores ranging from nm to μm scales and with ultrathin walls consisting of mono- or few-layered BN membranes. Research showed that this 3D BN foam presented super strong adsorbent ability in removing various oils and dyes^21^. Li *et al*. developed a kind of novel NaOH-embedded 3D porous BN (NaOH-3D BN) that was composed of vertically aligned and uniform nano flakes with high and hierarchical porosities. The NaOH-3D BN was envisaged to be practically valuable for indoor air purification because of its excellent removal performance for HCHO^22^.

Various adsorbents have been explored for the removal of hazardous organic dyes, oils pollution and heavy metal ions from aqueous solution, including the commonly used activated carbon, zeolite, mesoporous alumimum oxide, carbonaceous nanofiber adsorbents, magnetic powder composite, montmorillonite and so on^23–26^. Borah *et al*. synthesized porous carbon with high surface area from different tea precursors under thermochemical condition and the maximum adsorption capacity for methylene blue was found to be 402.25 mg g^−1 ^^27^. Li *et al*. developed a nanocomposite based on lignin grafted carbon nanotubes (L-CNTs) as a new type of adsorbent for water remediation. The large surface area and 3D structure enable the L-CNTs shows a high removal efficiency for Pb(II) (235 mg g^−1^) and oil droplet (200 mg L^−1^) from water^28^. In the future work, the efficient and high-quality adsorbents are still increasing required.

**Figure 1:**
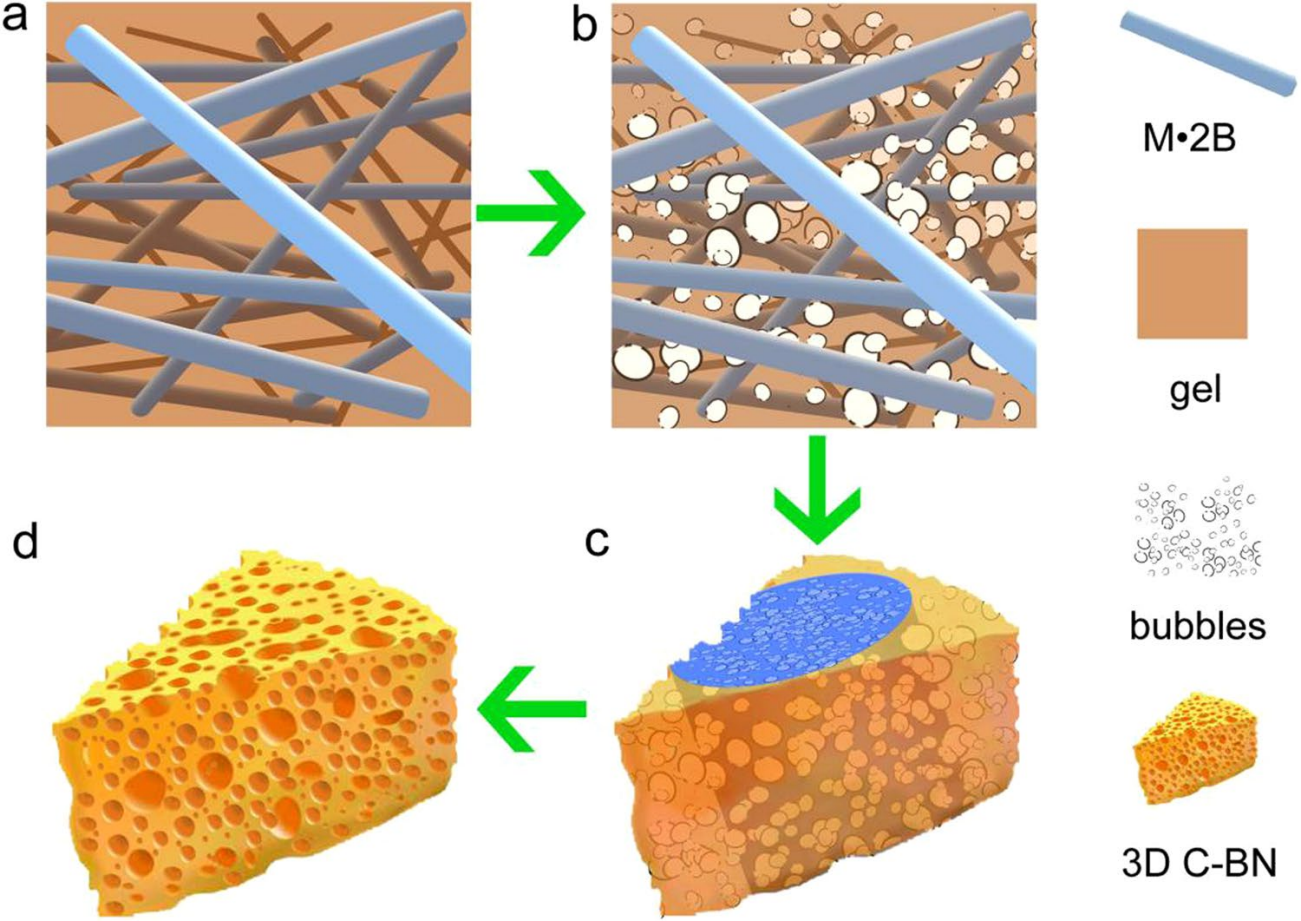
Diagrammatic sketch of the 3D C-BN synthesis mechanism.

In this contribution, we report an innovative method to synthesize a novel carbon-boron nitride (C-BN) composite material with unique cheese-like 3D morphology. The synthesis mechanism is illustrated by Fig. 1. Owing to the unique physical and chemical properties, we systematically investigated its adsorption abilities for different dyes, oils and metal ions in waste water. In addition, the adsorption capacity was compared with those of traditional powdered materials. The regenerative capacity after repeated adsorption of dyes and oils was finally studied.

## Results and Discussion

**Figure 2:**
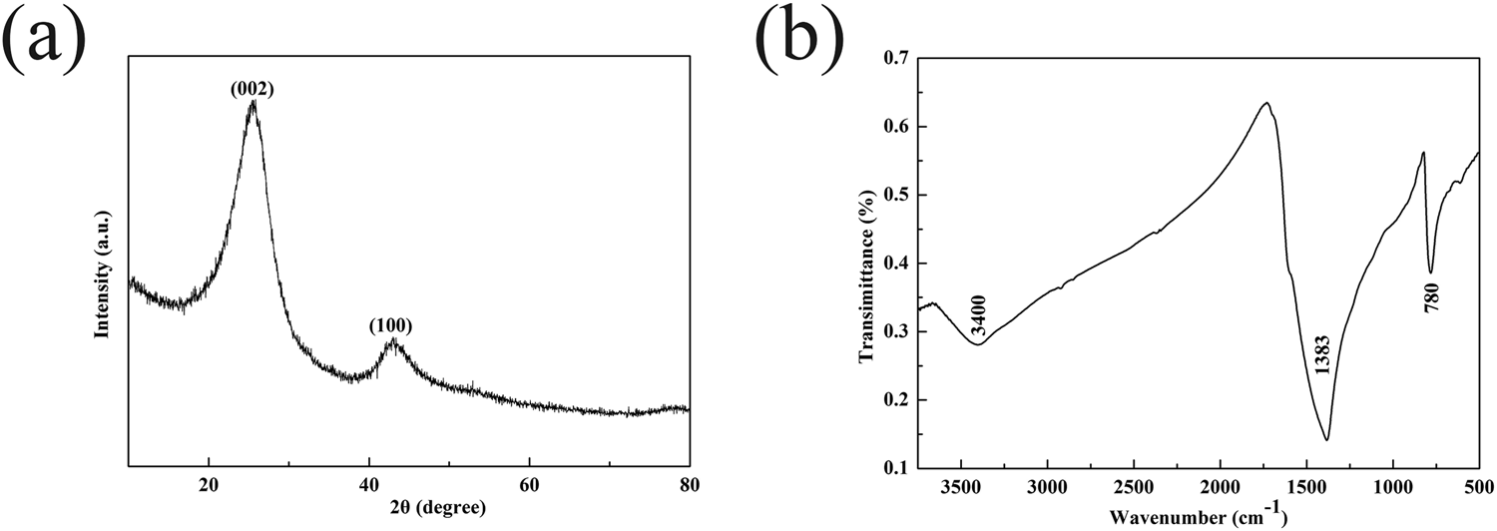
(**a**) XRD pattern and (**b**) FTIR spectra of 3D C-BN.

According to the XRD pattern (Fig. 2a), it can been seen that the broad diffraction peaks at ~24° and ~43° correspond to the (002) and (100) lattice planes of hBN (JCPDS No.34-0421) and graphite (JCPDS No. 65-6212)^19^, respectively. The FTIR spectrum of 3D C-BN is depicted in Fig. 2b. The main characteristic peaks of B-N (~1400 cm^−1^) and B-N-B (~800 cm^−1^) are both detected, indicating the main crystalline structures of hexagonal BN are existed. Meanwhile, the additional surface bands O-H (~3400 cm^−1^) is relatively weaken, suggesting the degree of structure ordering is reinforced by the addition of carbon. The results demonstrate that the sample is a composite of graphite and hBN with abundant surface functional bonds such as -OH which contribute to the adsorption performance.

In order to accurately obtain the compositions of C, N and O elements in the samples, the corresponding data of elemental analyzers are given in Table 1. As demonstrated above, the carbon content reaches 34.4 wt%, confirming that the composite materials with high carbon content have been successfully prepared.

**Table 1:** The contents of C, N and O.

Sample	C (wt%)	N (wt%)	O (wt%)
3D C-BN	34.4	15.08	22.2

**Figure 3:**
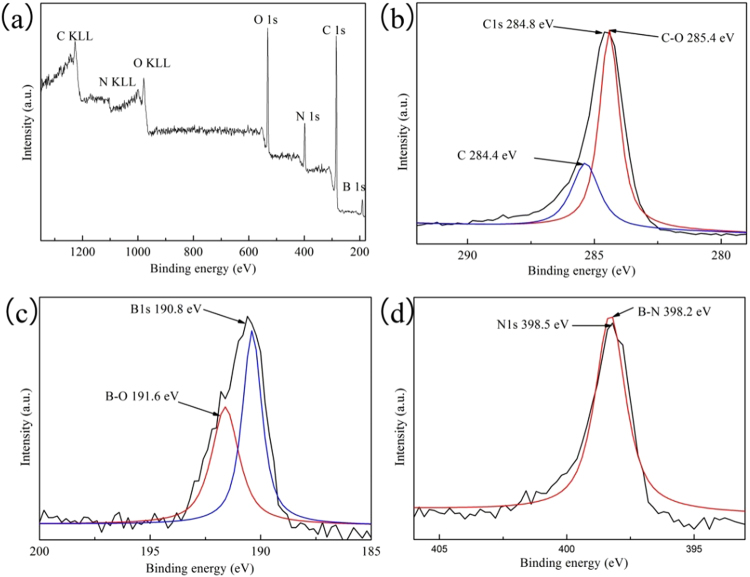
(**a**) XPS survey spectra of 3D C-BN; (**b**–**d**) High-resolution XPS spectra of C1s, B1s and N1s of 3D C-BN.

Furthermore, XPS spectra were also measured to identify the chemical composition and bonding state of the sample. The 3D C-BN sample is mainly composed of B, N, C and O. Figure 3b shows the C1s XPS spectrum that can be fitted into two peaks at 284.4 eV and 285.4 eV. The primary peak located at 284.4 eV is attributed to C–C bond, and the other could be assigned to C–O and graphitic carbon bonds. The B1s and N1s peaks located at 190.8 eV and 398.2 eV are shown in Fig. 3c,d. The peak at 191.6 eV in B1s spectrum is due to the existence of B-O bonds originating from the oxidation of B atoms on the surface or its precursor (B_2_O_3_)^29^. The results of B and N match with the previously reported values of h-BN^30^.

**Figure 4:**
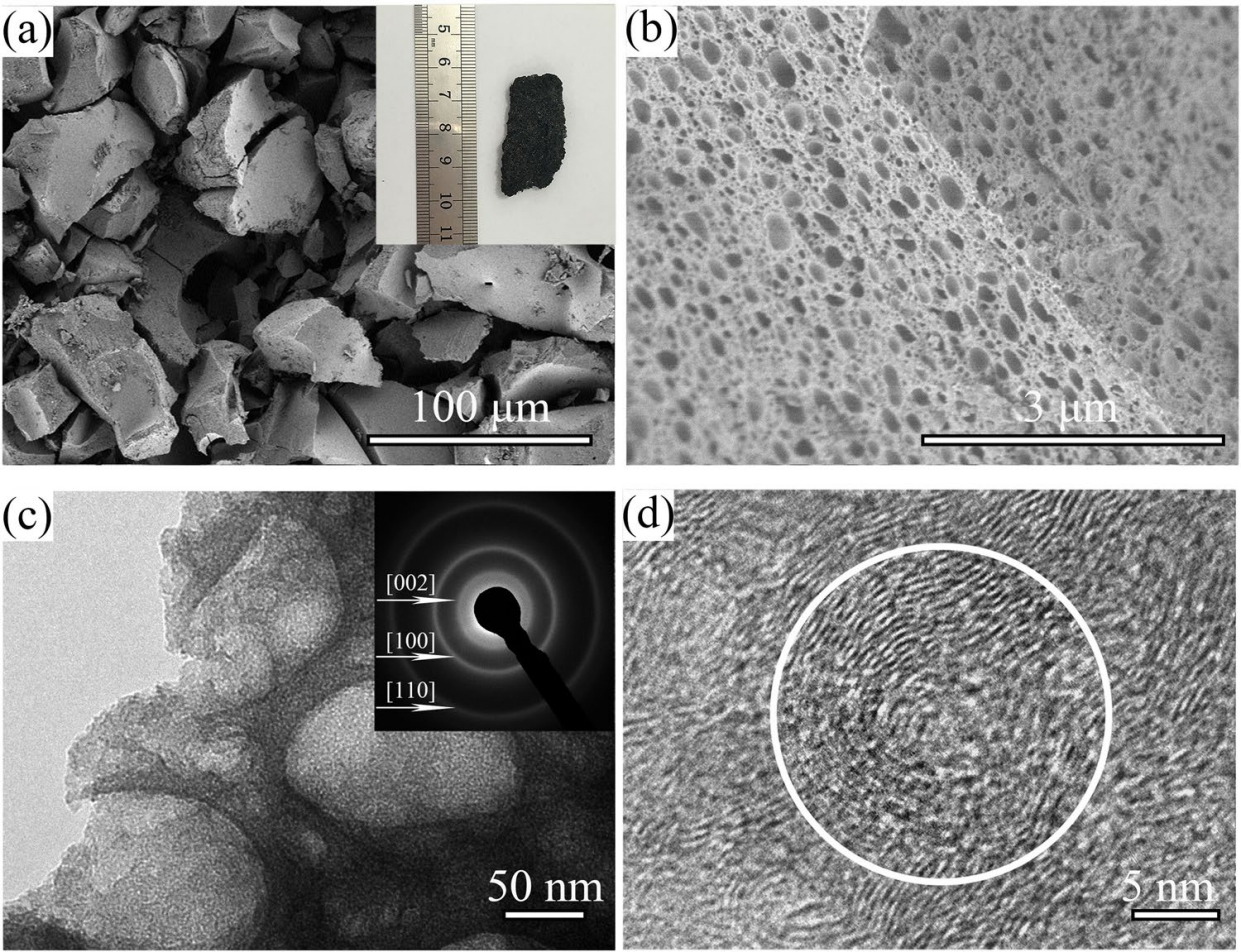
(**a**) Low-magnification SEM image and photographic image (inset); (**b**) High-magnification SEM image; (**c**) TEM image and SAED pattern (inset); (**d**) HRTEM image of 3D C-BN samples.

From the insert of Fig. 4a, the 3D C-BN material exhibits gray-black spongy macro appearance which can maintain its shape under a decent strength of 18.14 N (Fig. S1a). Even further, the sample is still able to bear a compressive strength of 11.73 N after immersion in water (Fig. S1b). The great advantage will make the material more convenient to use in water purification. Typical SEM images were taken in order to study the morphology of product. The 3D C-BN is composed of three dimensional bulks of a large size from 10 μm to 100 μm (Fig. 4a). Interestingly, the surface and cross-section of the bulks are covered with many dense holes. The unique structure we called cheese-like 3D BN. The pore sizes are in the range of 2~100 nm, which have been clearly displayed in Fig. 4b,c. The insert corresponding selected area electron diffraction (SAED) pattern reveals hexagonal structure of BN and carbon further. Figure 4d is the HRTEM image of an inner hole at the edge of one granule. The average distance of parallel adjacent fringes is around 0.35 nm, which matches well with the (002) parameter of hBN and graphite. Attractively, the lattice planes growing in a certain orientation seem like the structure of the onion, and the thickness of the onion skin is about 5 nm.

**Figure 5:**
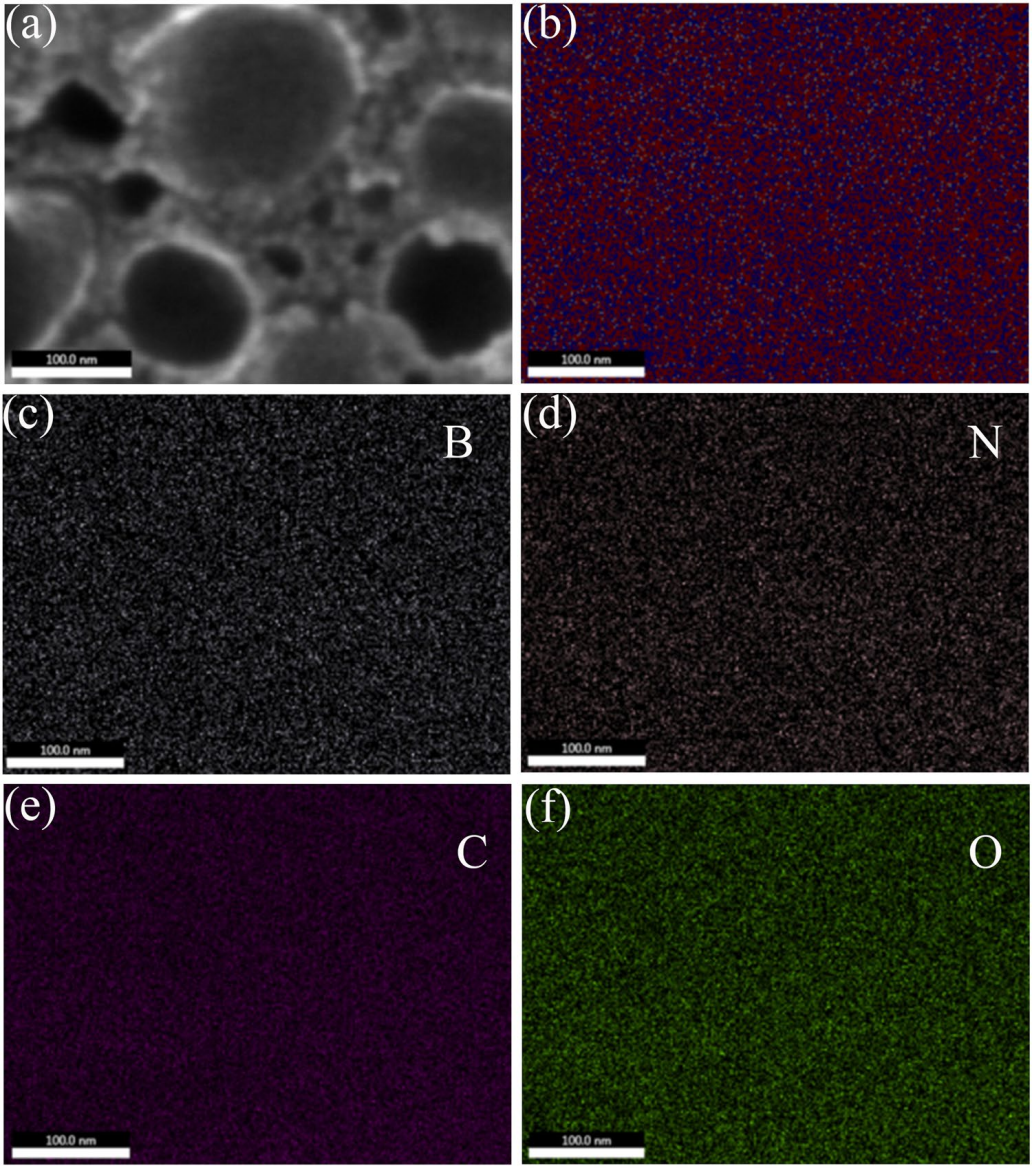
(**a**) The SEM image of 3D C-BN; (**b**) An overlay of B, N, C and O elemental mappings; (**c**–**f**) The individual spatially resolved distribution of B, N, C and O species.

The chemical compositions were characterized by spatially resolved elemental mapping based on the SEM of Fig. 5. The results clearly indicate that each element exhibits homogeneous distribution. Individual B, N, C and O elemental distributions (Fig. 5c–f) confirm that B, N and C are in the same crystal lattice. In addition, the adding of uniformly distributed O can enhance the adsorption capacity of 3D C-BN^31,32^.

**Figure 6:**
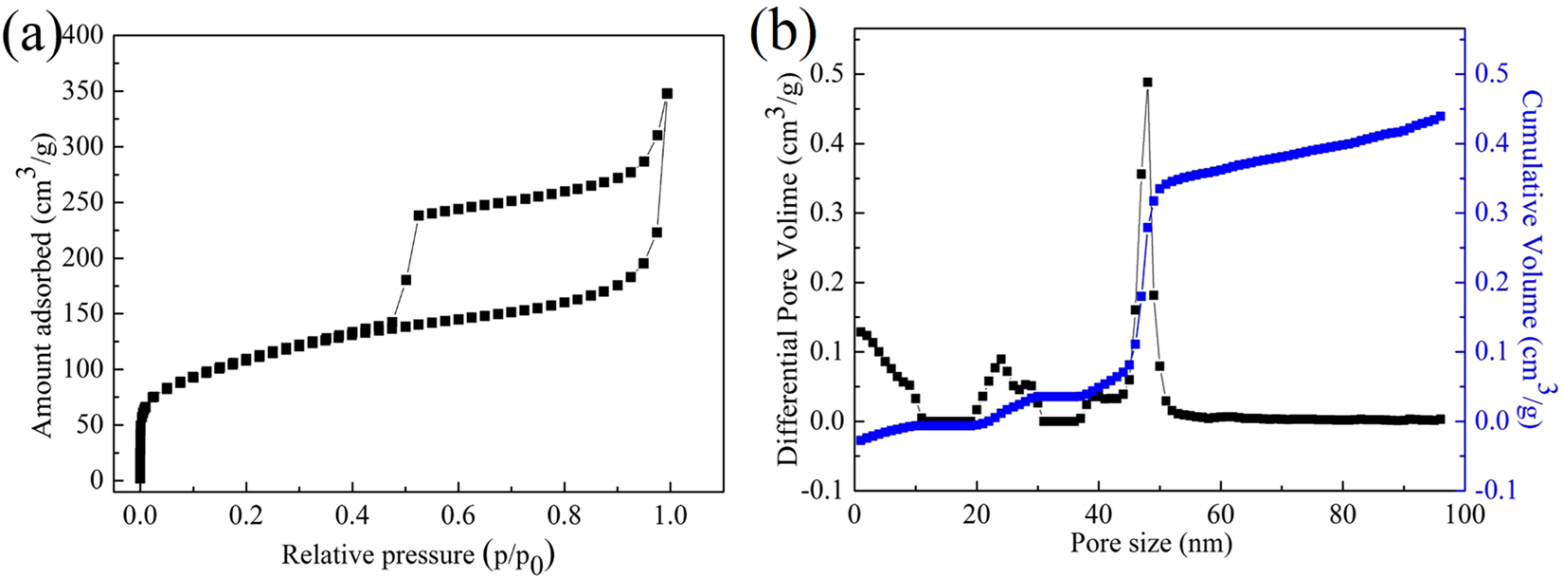
(**a**) Nitrogen adsorption/desorption isotherm; (**b**) The corresponding pore size distributions obtained by DFT method (black) and the cumulative pore size distribution (blue).

Figure 6a shows the nitrogen adsorption / desorption isotherms of the 3D C-BN sample. The measured isotherms illustrate a typical I type sorption behavior with the hysteresis loops belonged to type H4^33,34^. The specific surface area of the sample is 344.1 m^2^ g^−1^ and the high pore volume is 0.5 cm^3^ g^−1^ calculated by the framework of non-local density functional theory (NLDFT) method. The pore diameter distribution curve of sample obtained from the desorption branch reveals that the majority of pores are mainly about 48 nm and there is a narrow pore distribution from 45 to 52 nm, which can be ascribed to mesopores formed by the gas overflow during pyrolysis of precursors. Therefore, similar to activated carbon’s typical structure, the 3D C-BN samples possess hierarchical porous structure, which has considerable potential applications in adsorption, separation and catalysis^35^.

**Figure 7:**
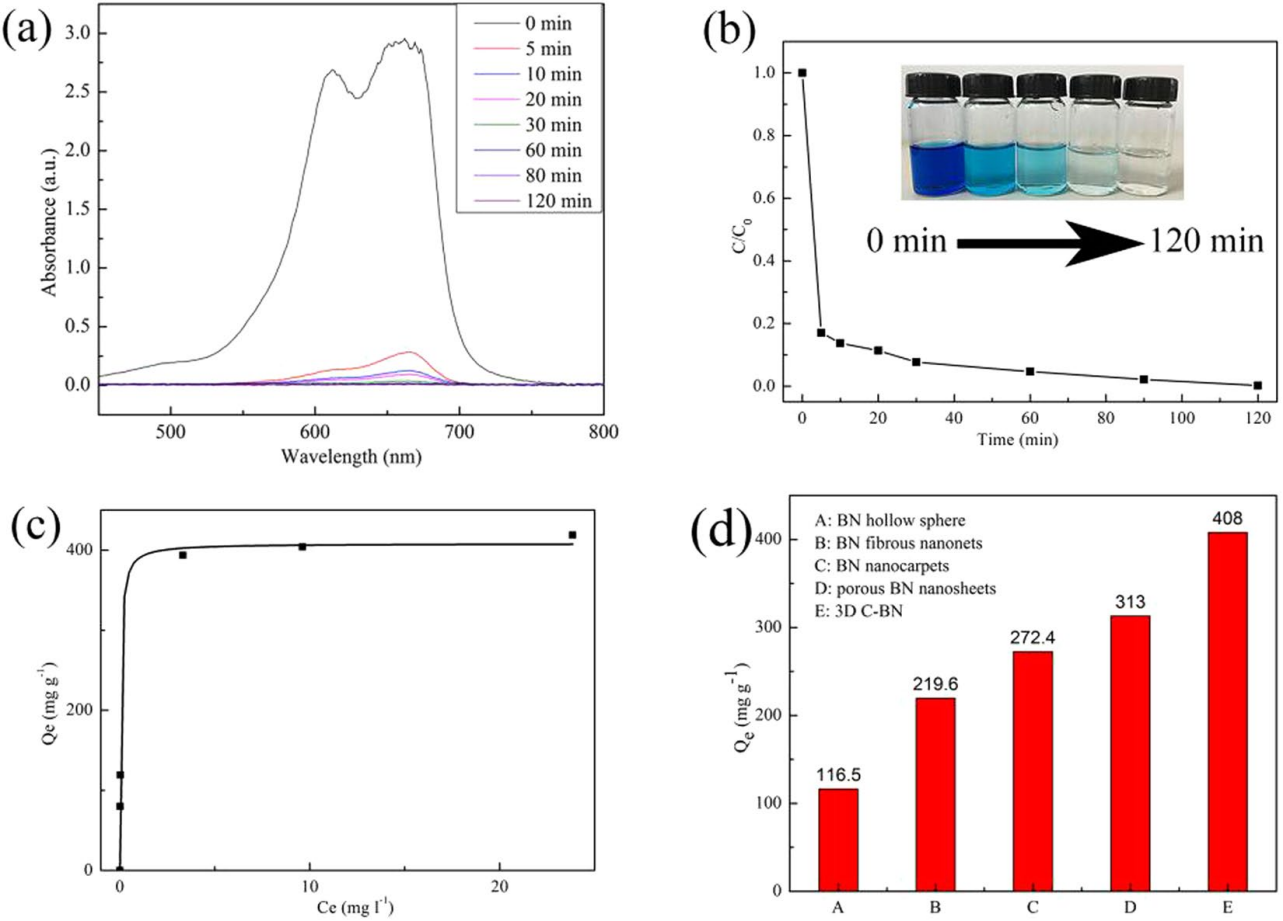
(**a**) UV-Vis adsorption spectra of aqueous MB solution treated by 3D C-BN; (**b**) The corresponding adsorption rate; (Inset) The images of the aqueous MB solution (50 mg l^−1^, 200 ml) at different time intervals by 100 mg 3D C-BN; (**c**) Adsorption isotherms of MB fitted by Langmuir model; (**d**) Different MB adsorption capacities of 3D C-BN with various BN materials.

Organic dyes have been considered as the main source of the water pollution. Removals of organic dyes from waste water have attracted more and more attention. We studied the adsorption performance of as-prepared 3D C-BN for the organic dye, i.e. MB from its aqueous solution. Figure 7a shows the UV-Vis absorption spectra of MB solution treated by 3D C-BN at different time intervals. Meanwhile, the corresponding adsorption rate is shown in Fig. 7b. Obviously, more than 88 wt% of MB was quickly removed from the solution at room temperature within 5 min, and over 99 wt% was adsorbed in 2 h. The inset photograph in Fig. 7b shows the change in colour of the solution at different times after adding of 3D C-BN. Figure 7c shows the adsorption isotherm of MB and the Langmuir model fits the experimental data well with the correlation coefficient higher than 0.99. The maximum adsorption capacity of 3D C-BN is 408.0 mg g^−1^, much higher than the previously reported BN materials such as BN hollow spheres (116.5 mg g^−1^), BN fibrous nanonets (219.6 mg g^−1^), BN nanocarpets (272.4 mg g^−1^) and BN porous nanosheets (313 mg g^−1^) (Fig. 7d)^12,36–38^.

**Figure 8:**
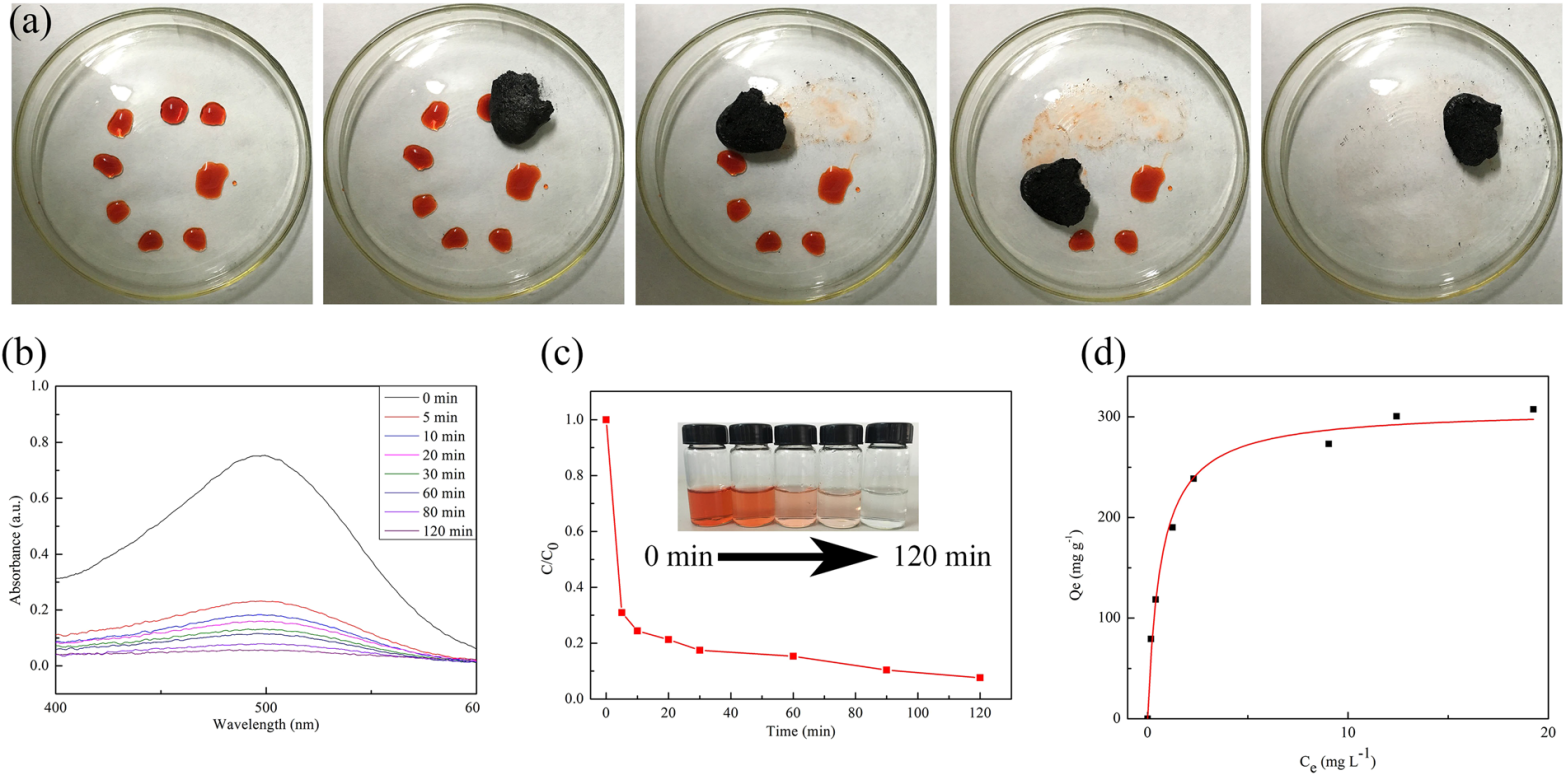
(**a**) The effective and rapid adsorption ability of the sample immersed CR solution within 1 min; (**b**) UV-Vis adsorption spectra; (**c**) The corresponding adsorption rate; (Inset) The images of the aqueous CR solution (50 mg l^−1^, 200 ml) at different time intervals after adding 100 mg 3D C-BN; (**d**) Adsorption isotherms of CR fitted by Langmuir model.

The 3D C-BN not only can effectively adsorb cationic dyes, but also adsorb anionic dyes. Figure 8a demonstrates that the sample can remove CR without destroying the original appearance. Figure 8b,c show the UV-Vis absorption spectra of the CR solution treated with 3D C-BN at different time intervals and the corresponding adsorption rate. 93 wt% of CR was largely adsorbed within 2 h at room temperature. The obtained maximum adsorption capacity is 307.6 mg g^−1^ and the correlation coefficient of the adsorption isotherm fitted by Langmuir model is more than 0. 99.

**Figure 9:**
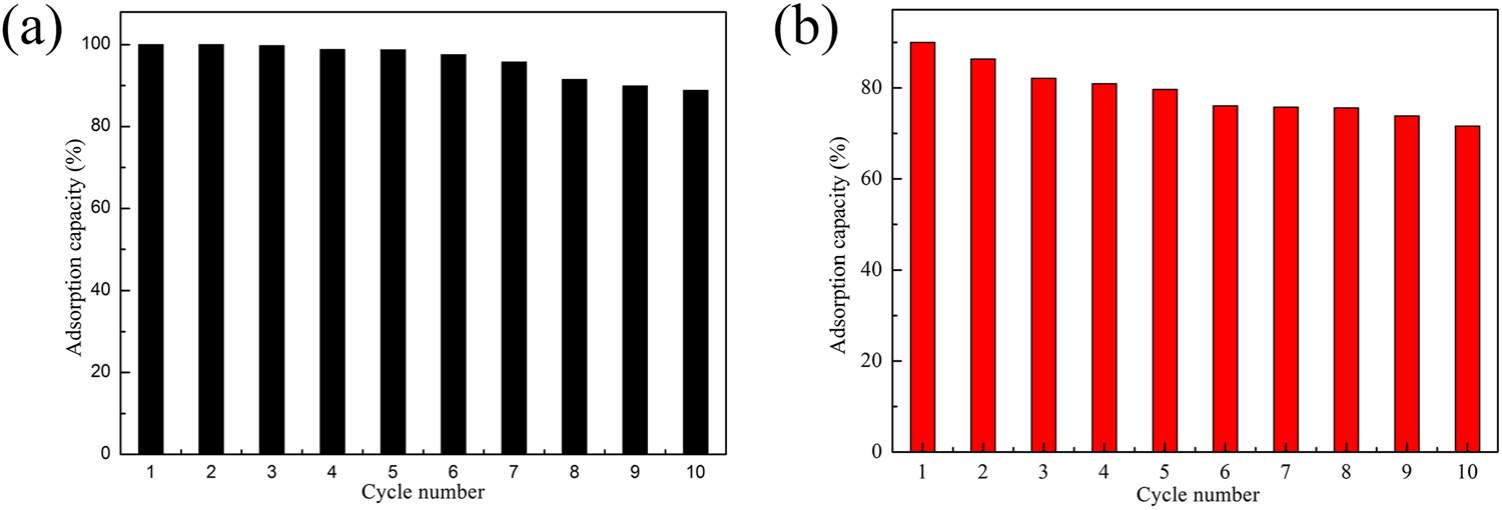
(**a**) MB and (**b**) CR adsorption recyclability of 3D C-BN.

Most importantly, the 3D C-BN sample can be reused in terms of dyes adsorption. The used 3D C-BN sample was heated at 500 °C in air for 2 h. After 10 times recycling, the adsorption capacity of 3D C-BN was still maintained 88.84% for MB and 71.61% for CR (Fig. 9a,b), indicating high recovery efficiencies.

**Figure 10:**
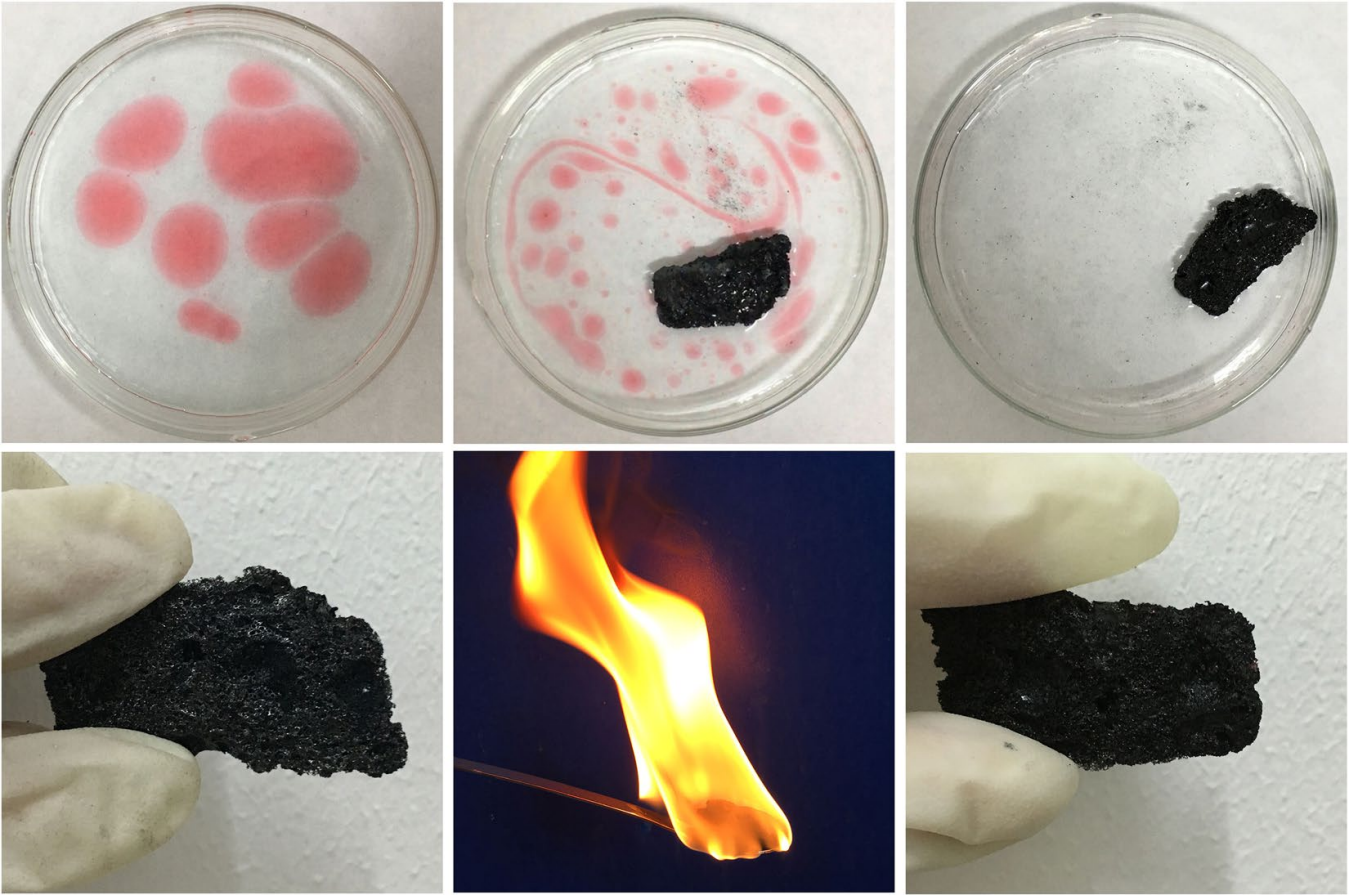
(**a**–**c**) The process of adsorbing gasoline by 3D C-BN; (**d**) Original appearance of the 3D C-BN; (**e**) Burning the sample after absorbing gasoline; (**f**) The appearance of 3D C-BN after six times recycling.

Figure 10a–c show different steps of the adsorbing gasoline process by using 3D C-BN. In Fig. 10a, the suspended red spots were the gasoline signed by the oil-soluble dyes. One block of 3D C-BN was added into the culture dish. All gasoline had been taken up by the sample within 5 minutes, which was still floating on the cleaned water (Fig. 10c). Moreover, the material could be reused several times by directly burning it in air to remove the adsorbed organics (Fig. 10e). After six times recycled utilization, the sample still maintains the same spongy appearance as before (Fig. 10f).

**Figure 11:**
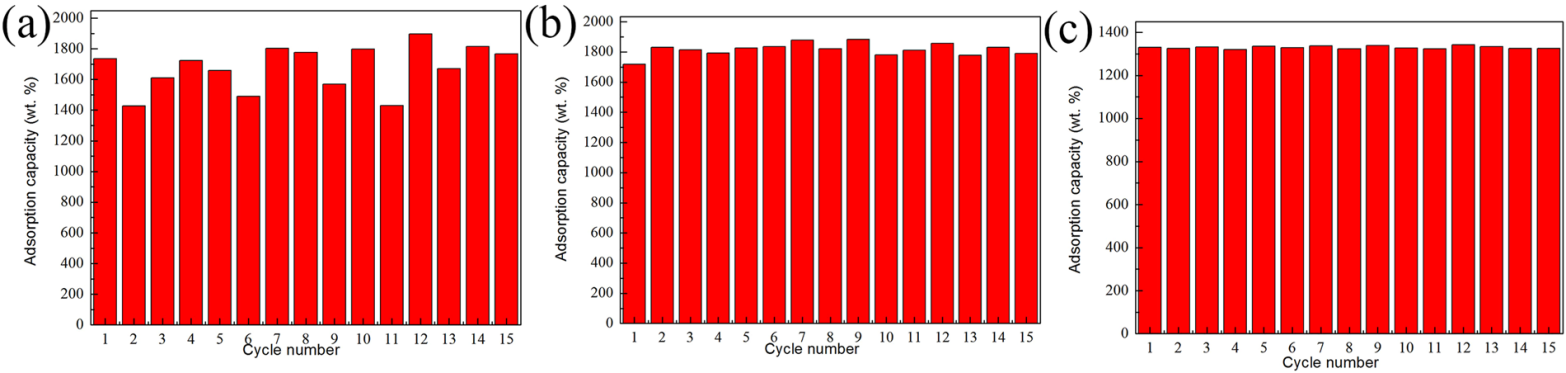
The recycle ability of 3D C-BN for (**a**) salad oil; (**b**) gasoline and (**c**) pump oil.

The recycle ability of 3D C-BN for multiple oils was studied by at least 15 cycles’ testing in Fig. 11. The method to remove gasoline is lit directly, while removing salad or pump oil are heating at 800 °C for 3 h. As shown in Fig. 11a, the first cycle of the sample for salad oil is about 1700 wt %, and there is still ~1500 wt% of adsorption capacity after 15 times recycling. The average adsorption capacity for salad oil is about 1600 wt%, which is twice as much as that of BN-based porous monoliths^39^. Figure 11b shows that the capacity for gasoline is about 1700 wt% for the first cycle and ~1800 wt% for the fifteenth cycle. For pump oil, the average adsorption capacity of ~1300 wt% is more stable, much higher than activated carbon and commercial bulk BN^40,41^.

**Figure 12:**
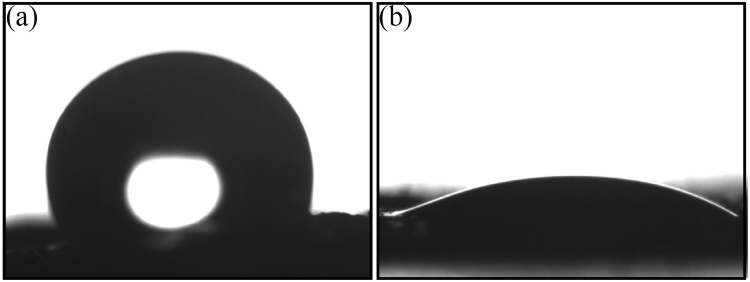
The contact angle of 3D C-BN bulk for (**a**) water, (**b**) salad oil.

It is noteworthy that the bulk samples float on the solution without water infiltration during the whole adsorbing process, which is attributed to the hydrophobic and lipophilic properties (Fig. 12). The contact angle of the water droplet on the sample bulk is ~112.1° (Fig. 12a), while that of oil is ~20.8° (Fig. 12b). The advantages of non-toxic, reusable, floatable and the bulk shape make the 3D C-BN a suitable candidate for oil pollution treatment.

**Figure 13:**
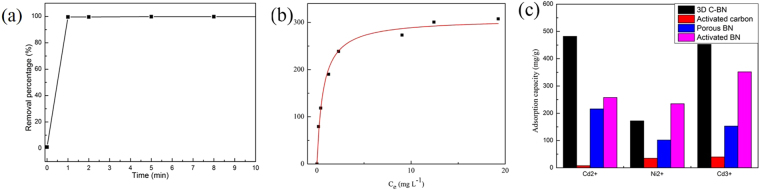
(**a**) Adsorption rate of the Cr^3+^ ions. (**b**) The corresponding adsorption isotherm fitted by Langmuir model. (**c**) Different adsorption capacities of 3D C-BN, the activated carbon, porous BN and activated BN for Cr^3+^, Ni^2+^ and Cd^2+^, respectively.

The pollution of heavy metal ions are harmful effects to human life and ecological environment, thus has become one of the most serious environmental problems in the last decades. The 3D C-BN with polar B-N bonds is an ideal material for removing metal ions^42^. 100 mg of 3D C-BN was added into 200 mL aqueous solution with the initial Cr^3+^ concentration of 50 mg/L for Cr^3+^ adsorption at room temperature (pH = 5.5). Figure 13a illustrates the fast adsorption rate of 99 wt% within 2 min for removing Cr^3+^, much quicker than the reported activated boron nitride^14^. After 1 h, Cr^3+^ in aqueous solution was almost completely removed. According to the experimental data, the adsorption capacity of 3D C-BN for Cr^3+^ is higher than that of activated carbon as well as activated boron nitride^43,44^. The corresponding adsorption isotherm is shown in the Fig. 13b. The Langmuir model well fits with the experimental data with the correlation coefficient of 0.999 and the maximum adsorption capacity of Cr^3+^ is calculated to be 453.1 mg g^−1^. Moreover, the detected maximum uptake capacities of Cd^2+^ and Ni^2+^ are 482.1 and 172.6 mg g^−1^, respectively. (The adsorption rates of Cd^2+^, Ni^2+^ are given in Fig. S2, corresponding adsorption isotherms are given in Fig. S3). Comparison of adsorption capacities of 3D C-BN, activated carbon, porous BN and activated BN for the heavy metal ions under the same condition (Fig. 13c), 3D C-BN exhibited distinct advantages and strong practicability.

## Conclusions

In summary, a cheese-like 3D C-BN was successfully synthesized by a simple heated treatment without any templates. The obtained sample has three-dimensional porous cheese-like structure with bulk size ranging from 10 to 100 μm. It is noteworthy that the surface and cross-section of the sample are richly supplied with 5~100 nm sized pores. Due to its abundant pore structure, various surface function groups and polar B-N bond, the 3D C-BN can match up with the all-powerful adsorbent materials for various kinds of pollutants. Significantly, it can effectively adsorb dye pollutions (MB and Cr) and oils (salad oil, gasoline and pump oil), as well as the most harmful heavy metal ions (Cd^2+^, Ni^2+^ and Cr^3+^) in aqueous solution. In contrast to the other adsorbents, the adsorption performance of 3D C-BN is more superior. The product can be recycled with a high adsorption ability remained, which is consistent with the trend of energy conservation. Hence, our work illustrated the great potential of 3D C-BN material in water treatment.

## Methods

### Preparation of 3D C-BN

3D C-BN was prepared by a simple heated treatment with two different precursors. Firstly, 6.96 g of boron trioxide (B_2_O_3_) and 9.95 g of triethanolamine (C_6_H_15_NO_3_) were uniformly mixed in a hydrothermal reactor and heated to 200 °C for 10 h to obtain the brown precursor gel *via* solvothermal process.

Secondly, 3.71 g of H_3_BO_3_ and 3.78 g of C_3_N_6_H_6_ were dissolved in 250 g of distilled water. The reaction mixtures were heated at 85 °C for 12 h, and then naturally cooled to room temperature. The obtained white precipitate was filtered and washed with deionized water. The samples were dried at 90 °C for 12 h to obtain fibrous melamine diborate (M·B2) crystals^45^.

At last, M·B2 was added into the as-prepared precursor gel (quality ratio = 1:1), gluing with each other tightly *via* chemical bond and hydrogen bond (Fig. 1a). The mixture was treated at 1100 °C (5 °C min^−1^) for 4 h in a flow of N_2_ (0.8 L min^−1^). In the heat treatment process, a large number of bubbles were formed by pyrolysis of precursor gel (Fig. 1b). With the increase of temperature, the M.B2 fibers as the support of 3D structure begun to pyrolysis and generated more bubbles (Fig. 1c). The gases in the bubbles overflowed and formed foam structure containing of numerous pores (Fig. 1d). After heat treatment, the 3D C-BN samples were finally prepared.

### Adsorption and removal of dyes and metal ions

In this section, 3D C-BN bulk samples were ground into powder samples as adsorbent. MB (C_16_H_18_ClN_3_S), CR (C_32_H_22_N_6_Na_2_O_6_S_2_), CrCl_3_.6H_2_O, CdCl_2_.2.5H_2_O and NiCl_2_.6H_2_O were dissolved in water and adjusted to the required concentration. The pH values of the initial solutions were adjusted from 5.5 to 6.0 by adding of 0.1 M HNO_3_ solution. The pH value of optimum adsorption for MB, CR, Cr^3+^, Cd^2+^, Ni^2+^ was 6.0, 6.0, 5.5, 5.5, 6.0, respectively^46,47^. A series of initial concentrations were carried out by the adsorption tests to obtain the isotherms. The removal percentages of pollutants were calculated by the following formula:

1 $$\eta ( \% )=({C}_{0}-{C}_{e})\times 100/{C}_{0}$$

where *C*_0_ and *C*_*e*_ (mg L^−1^) are the initial concentration and equilibrium concentration, respectively. *η* is the removal percentage of the pollutants.

The Langmuir adsorption isotherm was used to represent the relationship between the adsorption capacity of adsorbent (*Q*_*e*_, mg g^−1^) and the equilibrium concentration of adsorbate (*C*_*e*_, mg L^−1^), the Langmuir isotherm equation is shown below:

2 $${Q}_{e}={Q}_{m}\times K\times {C}_{e}/(1+K\times {C}_{e})$$

where *Q*_*m*_ (mg g^−1^) is the maximum adsorption capacity corresponding to complete monolayer covering on the adsorbents and *K* (l mg^−1^) is the equilibrium constant related to the affinity of binding sites.

### Adsorption of oils

In this section, 3D C-BN samples were cut into about 500 mg bulk as adsorbent.

The adsorption capacity (*C*) was computed by Eq. 3:

3 $$C=({M}_{a}-{M}_{0})/{M}_{0}$$

where *M*_0_ and *M*_*a*_ are the weights before and after adsorption, respectively.

### Characterization

X-ray photoelectron spectroscopy (XPS) was examined by a VGESCALAB 210 electron spectrometer. The microscopic structure of samples was investigated by X-ray powder diffraction (XRD, D8 Focus, Bruker) analysis. Meanwhile, the Fourier transformer infrared (FTIR) spectra were recorded on a Nicolet 7100 spectro photometer between 400 and 4000 cm^−1^ (The samples are ground to powder, mixed with anhydrous potassium bromide and pressed into a film with thickness of 0.57 mm). Conventional elemental analyzers (TC500 and CS230, Leco) were used to analyze the detailed O, N, and C contents. The bulk mechanic properties were determined by using a tension test machine (SHIMADZU EZ-S, Japan), and the testing sample was cut into about 1~2 cm^3^ cubic. The morphology and structure of 3D C-BN were studied by scanning electron microscope (SEM, S-4800, Hitachi) as well as the transmission electron microscopy (TEM, Tecnai F20, Philips). The nitrogen physisorption isotherms were measured at −196 °C on an AutoSorb iQ-C TCD analyzer. Prior to the measurement, the 3D C-BN was activated in a vacuum at 300 °C for 3 h. The Brunauer-Emmett-Teller (BET) specific surface area was calculated from the nitrogen adsorption data in the relative pressure ranging from 0.01 to 0.3. The solution concentrations of the MB and CR were measured by a double beam UV/vis spectrophotometer (U-3900H, Hitachi) and the concentrations of the Cr^3+^, Cd^2+^ and Ni^2+^ were determined by the high dispersion inductively coupled plasma emission spectroscopy (ICP) (Teledyne-Leeman Labs, USA).

## Electronic supplementary material

supplementary material
