# Supplementary material for: Novel multifunctional cheese-like 3D carbon-BN as a highly efficient adsorbent for water purification
Source: Sci Rep. 2018 Jan 18;8:1104. doi: 10.1038/s41598-018-19541-5 (PMC5773703; doi:10.1038/s41598-018-19541-5)
Supplement: Supplementary file 1 — supplementary material [file 41598_2018_19541_MOESM1_ESM.pdf]

Supplementary Material

Novel multifunctional cheese-like 3D carbon-BN as a highly efficient adsorbent for water purification

Zhenya Liu [1, 2], Yi Fang [1, 2, *], Huichao Jia [1, 2], Chong Wang [1, 2], Qianqian Song [1, 2], Lanlan Li [1, 2], Jing Lin [1, 2], Yang Huang [1, 2], Chao Yu [1, 2] & Chengchun Tang [1, 2]

[1] *School of Materials Science and Engineering, Hebei University of Technology, Tianjin 300130, P. R. China.*

[2] *Hebei Key Laboratory of Boron Nitride Micro and Nano Materials, Hebei University of Technology, Tianjin 300130, P. R. China.*

* Correspondence and requests for materials should be addressed to Y.F. (e-mail: kerrfy@126.com)

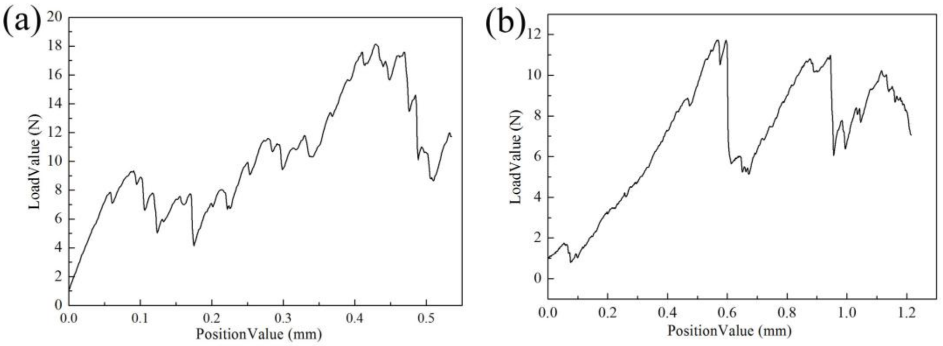

Fig S1. The stress-strain curves of 3D C-BN (a) and 3D C-BN during the repeated water purification processes (b)

Fig. S1a shows the stress-strain curves. With the movement of position 3D C-BN maintain a decent compressive strength of 18.14 N (the bulk of 3D C-BN is 1778.74 mm^3). The 3D C-BN materials have enough mechanical strength that can maintain the strength of 11.73 N during the repeated water purification processes (the bulk of recycled 3D C-BN is 1963.66 mm^3) showed in Fig. S1b. In Fig. S1, every downward peak means a part of collapsed hole. The bulk 3D C-BN is differentiated from

traditional powder adsorbents, which can bring more convenience in practical applications.

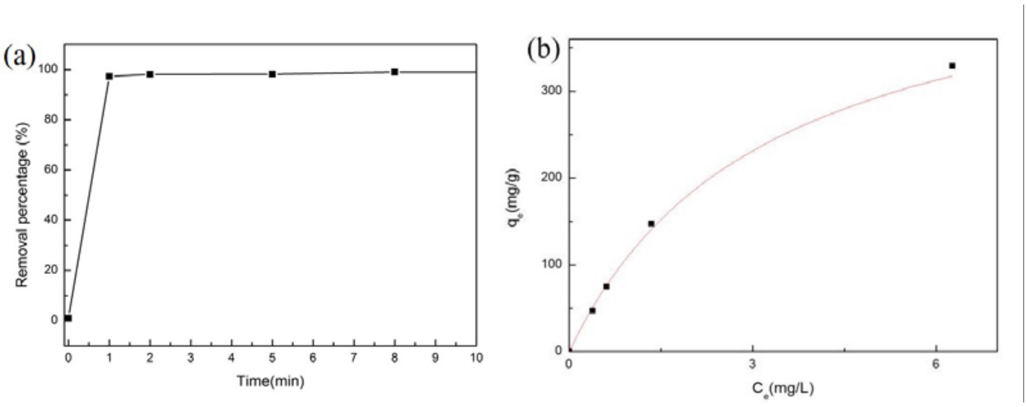

Fig S2. (a) Adsorption rate of the Cd^{2+} ions. (b) The corresponding adsorption isotherm fitted by Langmuir model.

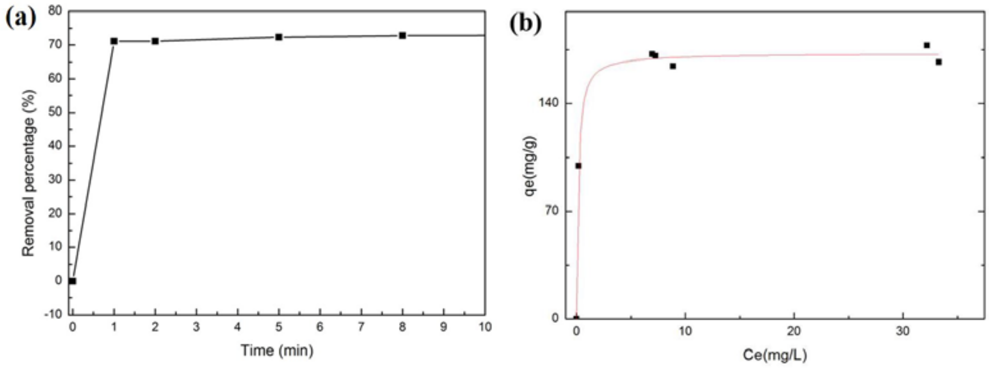

Fig S3. (a) Adsorption rate of the Ni^{2+} ions. (b) The corresponding adsorption isotherm fitted by Langmuir model.

Fig. S2a gives the hugely fast adsorption rate of 99 wt % within 2 min for removing Cd^{2+}. After 1 hour, the removal percentage getting ~ 100 wt%. Corresponding adsorption isotherm is shown in Fig. S2b. The Langmuir model well fits with the experimental data with the correlation coefficient of ＞0.99 and the maximum adsorption capacity of Cd^{2+} is 482.11 mg/g. As shown in Fig. S3a. More than 70 wt% of Ni^{2+} was removed within 2 mins. The corresponding adsorption isotherm is shown in the Fig. S3b. The Langmuir model well fits with the experimental data with the correlation coefficient of ＞0.99 and the maximum adsorption capacity of Ni^{2+} is 172.6 mg g^{-1}.
